# 3-{[3-(4-Meth­oxy­phen­yl)-4,5-dihydro-1,2-oxazol-5-yl]meth­yl}-1,5-dimethyl-1*H*-1,5-benzodiazepine-2,4(3*H*,5*H*)-dione

**DOI:** 10.1107/S1600536811022872

**Published:** 2011-06-18

**Authors:** Rachida Dardouri, Yousef Kandri Rodi, Natalie Saffon, El Mokhtar Essassi, Seik Weng Ng

**Affiliations:** a Laboratoire de Chimie Organique Appliquée, Faculté des Sciences et Techniques, Université Sidi Mohamed Ben Abdallah, Fés, Morocco; b Service Commun Rayons-X FR2599, Université Paul Sabatier Bâtiment 2R1, 118 route de Narbonne, Toulouse, France; c Laboratoire de Chimie Organique Hétérocyclique, Pôle de Compétences Pharmacochimie, Université Mohammed V-Agdal, BP 1014 Avenue Ibn Batout, Rabat, Morocco; d Department of Chemistry, University of Malaya, 50603 Kuala Lumpur, Malaysia

## Abstract

The mol­ecule of the title compound, C_22_H_23_N_3_O_4_, features a benzodiazepine fused-ring system whose seven-membered ring adopts a boat-shaped conformation (with the C atoms of the fused-ring as the stern and the methine C atom as the prow). The methyl­ene C atom connected to the methine C atom occupies an equatorial position. The methyl­ene C atom is connected to the five-membered oxazole ring, both of which are disordered over two positions in a 0.634 (4):0.366 (4) ratio. Weak inter­molecular C—H⋯O hydrogen bonding is present in the crystal structure.

## Related literature

For a related compound, 1,5-dimethyl-3-[(3-phenyl-4,5-dihydro-1,2-oxazol-5-yl)meth­yl]-1*H*-1,5-benzodiazepine-2,4(3*H*,5*H*)-dione, see: Dardouri *et al.* (2010 ▶).
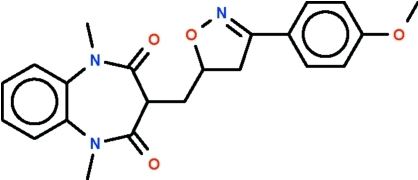


## Experimental

### 

#### Crystal data

C_22_H_23_N_3_O_4_
*M* *_r_* = 393.43
Monoclinic, 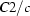

*a* = 28.0041 (5) Å
*b* = 15.4644 (3) Å
*c* = 9.0350 (2) Å
β = 93.235 (1)°
*V* = 3906.52 (13) Å^3^
*Z* = 8
Mo *K*α radiation
μ = 0.09 mm^−1^
*T* = 293 K
0.40 × 0.05 × 0.05 mm

#### Data collection

Bruker APEXII diffractometer
30861 measured reflections
3445 independent reflections
2548 reflections with *I* > 2σ(*I*)
*R*_int_ = 0.047

#### Refinement

*R*[*F* ^2^ > 2σ(*F* ^2^)] = 0.061
*wR*(*F* ^2^) = 0.179
*S* = 1.05
3445 reflections
272 parameters
41 restraints
H-atom parameters constrained
Δρ_max_ = 0.31 e Å^−3^
Δρ_min_ = −0.27 e Å^−3^

### 

Data collection: *APEX2* (Bruker, 2005 ▶); cell refinement: *SAINT* (Bruker, 2005 ▶); data reduction: *SAINT*; program(s) used to solve structure: *SHELXS97* (Sheldrick, 2008 ▶); program(s) used to refine structure: *SHELXL97* (Sheldrick, 2008 ▶); molecular graphics: *X-SEED* (Barbour, 2001 ▶); software used to prepare material for publication: *publCIF* (Westrip, 2010 ▶).

## Supplementary Material

Crystal structure: contains datablock(s) global, I. DOI: 10.1107/S1600536811022872/xu5243sup1.cif

Structure factors: contains datablock(s) I. DOI: 10.1107/S1600536811022872/xu5243Isup2.hkl

Supplementary material file. DOI: 10.1107/S1600536811022872/xu5243Isup3.cml

Additional supplementary materials:  crystallographic information; 3D view; checkCIF report

## Figures and Tables

**Table 1:** Hydrogen-bond geometry (Å, °)

*D*—H⋯*A*	*D*—H	H⋯*A*	*D*⋯*A*	*D*—H⋯*A*
C4—H4⋯O3^i^	0.93	2.51	3.367 (7)	154
C11—H11*B*⋯O1^ii^	0.96	2.56	3.501 (5)	168

Symmetry codes: (i) 
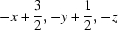
; (ii) 
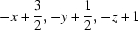
.

**Fig. 1.**
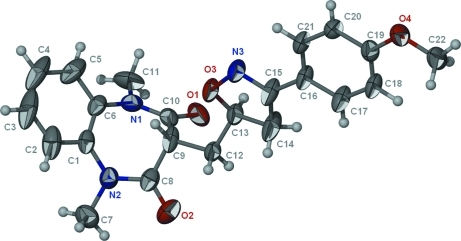
Thermal ellipsoid plot (Barbour, 2001) of C22H23N3O4 at the 50% probability level; hydrogen atoms are drawn as arbitrary radius. The disorder is not shown.

## supplementary crystallographic information

### Comment

A previous study reported the structure of 1,5-dimethyl-3-[(3-phenyl-1,2-oxazol-5-yl)methyl]-1,5-benzodiazepine-2,4-dione (Dardouri *et al.*, 2010). The phenyl group in this compound is replaced by an anisyl group in the title compound (Scheme I). The molecule of C_22_H_23_N_3_O_4_ features a benzodiazepine fused-ring whose seven-membered ring adopts a boat-shaped conformation (with the C atoms of the fused-ring as the stern and the methine C atom as the prow). The methylene C atom connected to the methine C atom occupies an equatorial position (Fig. 1).

### Experimental

To a solution of 3-allyl-1,5-dimethyl-1,5-benzodiazepine-2,4-dione (0.25 g, 1 mmol) and 4-methoxybenzaldoxime (0.2 g, 1.3 mmol) in chloroform (10 ml) was added at 0°C a solution of 24% bleach (4 ml). Stirring was continued for 4 h. The organic layer was dried over sodium sulfate and the solvent evaporated under reduced pressure. The residue was then purified by column chromatography on silica gel by using a mixture of hexane and ethyl acetate (1/1) as eluent. Colorless crystals were isolated when solvent was allowed to evaporate.

### Refinement

Carbon-bound H-atoms were placed in calculated positions (C—H 0.93–0.97 Å) and were included in the refinement in the riding model approximation, with *U*(H) set to 1.2–1.5*U*(C).

The oxazole ring and the methylene linkage connected to it are disordered over two positions in a 63.6 (1):36.4 ratio. The carbon–carbon distances were restrained to 1.50±0.01 Å; the pair of carbon–oxygen distances were restrained to 0.01 Å of each other, as were the pairs of carbon–nitrogen and nitrogen-oxygen distances. The temperature factors of the primed atoms were set to those of the unprimed ones, and the anisotropic temperature factors were restrained to be nearly isotropic.

### Crystal data

**Table:**

C_22_H_23_N_3_O_4_	*F*(000) = 1664
*M**_r_* = 393.43	*D*_x_ = 1.338 Mg m^−^^3^
Monoclinic, *C*2/*c*	Mo *K*α radiation, λ = 0.71073 Å
Hall symbol: -C 2yc	Cell parameters from 5272 reflections
*a* = 28.0041 (5) Å	θ = 2.6–21.8°
*b* = 15.4644 (3) Å	µ = 0.09 mm^−^^1^
*c* = 9.0350 (2) Å	*T* = 293 K
β = 93.235 (1)°	Prism, colorless
*V* = 3906.52 (13) Å^3^	0.40 × 0.05 × 0.05 mm
*Z* = 8	

### Data collection

**Table:**

Bruker APEXII diffractometer	2548 reflections with *I* > 2σ(*I*)
Radiation source: fine-focus sealed tube	*R*_int_ = 0.047
graphite	θ_max_ = 25.0°, θ_min_ = 1.5°
φ and ω scans	*h* = −33→31
30861 measured reflections	*k* = −18→18
3445 independent reflections	*l* = −10→10

### Refinement

**Table:**

Refinement on *F*^2^	Secondary atom site location: difference Fourier map
Least-squares matrix: full	Hydrogen site location: inferred from neighbouring sites
*R*[*F*^2^ > 2σ(*F*^2^)] = 0.061	H-atom parameters constrained
*wR*(*F*^2^) = 0.179	*w* = 1/[σ^2^(*F*_o_^2^) + (0.0764*P*)^2^ + 5.3445*P*] where *P* = (*F*_o_^2^ + 2*F*_c_^2^)/3
*S* = 1.05	(Δ/σ)_max_ = 0.001
3445 reflections	Δρ_max_ = 0.31 e Å^−^^3^
272 parameters	Δρ_min_ = −0.27 e Å^−^^3^
41 restraints	Extinction correction: *SHELXL97* (Sheldrick, 2008), Fc^*^=kFc[1+0.001xFc^2^λ^3^/sin(2θ)]^-1/4^
Primary atom site location: structure-invariant direct methods	Extinction coefficient: 0.0013 (3)

### Fractional atomic coordinates and isotropic or equivalent isotropic displacement parameters (Å^2^)

**Table:**

	*x*	*y*	*z*	*U*_iso_*/*U*_eq_	Occ. (<1)
O1	0.67566 (10)	0.19618 (16)	0.5304 (3)	0.0852 (8)	
O2	0.57207 (8)	0.11159 (17)	0.2866 (3)	0.0770 (7)	
O3	0.63939 (16)	0.3964 (3)	0.2387 (4)	0.0605 (14)	0.634 (4)
O4	0.54628 (8)	0.85003 (14)	0.3904 (3)	0.0710 (7)	
N1	0.71472 (9)	0.14146 (16)	0.3388 (3)	0.0578 (7)	
N2	0.63531 (8)	0.07484 (15)	0.1556 (3)	0.0536 (6)	
N3	0.6331 (2)	0.4866 (4)	0.2330 (7)	0.060 (2)	0.634 (4)
C1	0.67932 (11)	0.09694 (18)	0.0955 (3)	0.0523 (7)	
C2	0.68467 (17)	0.0844 (3)	−0.0552 (4)	0.0855 (12)	
H2	0.6590	0.0641	−0.1152	0.103*	
C3	0.7269 (3)	0.1015 (3)	−0.1157 (6)	0.119 (2)	
H3	0.7296	0.0928	−0.2167	0.143*	
C4	0.7656 (2)	0.1314 (3)	−0.0313 (7)	0.117 (2)	
H4	0.7946	0.1413	−0.0738	0.141*	
C5	0.76090 (14)	0.1467 (2)	0.1191 (5)	0.0880 (13)	
H5	0.7865	0.1691	0.1768	0.106*	
C6	0.71793 (11)	0.12863 (18)	0.1834 (3)	0.0542 (8)	
C7	0.61090 (12)	−0.0048 (2)	0.1047 (4)	0.0737 (10)	
H7A	0.5898	−0.0238	0.1783	0.111*	
H7B	0.6342	−0.0489	0.0892	0.111*	
H7C	0.5928	0.0064	0.0134	0.111*	
C8	0.61146 (11)	0.1295 (2)	0.2423 (3)	0.0554 (8)	
C9	0.63728 (13)	0.21183 (18)	0.2874 (4)	0.0650 (9)	
H9	0.6512	0.2368	0.1999	0.078*	0.634 (4)
H9'	0.6510	0.2375	0.2002	0.078*	0.366 (4)
C10	0.67758 (12)	0.18422 (18)	0.3971 (4)	0.0602 (8)	
C11	0.75503 (13)	0.1155 (3)	0.4405 (5)	0.0895 (12)	
H11A	0.7430	0.0928	0.5300	0.134*	
H11B	0.7749	0.1648	0.4635	0.134*	
H11C	0.7735	0.0718	0.3942	0.134*	
C12	0.59677 (16)	0.2732 (3)	0.3369 (6)	0.0481 (11)	0.634 (4)
H12A	0.5712	0.2755	0.2599	0.058*	0.634 (4)
H12B	0.5836	0.2506	0.4261	0.058*	0.634 (4)
C13	0.61609 (16)	0.3625 (3)	0.3660 (5)	0.0512 (10)	0.634 (4)
H13	0.6381	0.3630	0.4544	0.061*	0.634 (4)
C14	0.57479 (7)	0.42592 (11)	0.3838 (2)	0.0855 (13)	0.634 (4)
H14A	0.5455	0.4069	0.3312	0.103*	0.634 (4)
H14B	0.5690	0.4365	0.4870	0.103*	0.634 (4)
C15	0.59627 (7)	0.50273 (11)	0.3118 (2)	0.0683 (10)	
C16	0.58267 (7)	0.59290 (11)	0.3351 (2)	0.0551 (8)	
C17	0.54373 (7)	0.61376 (11)	0.4159 (2)	0.0683 (10)	
H17	0.5262	0.5696	0.4569	0.082*	
C18	0.53026 (11)	0.6984 (2)	0.4372 (3)	0.0610 (8)	
H18	0.5038	0.7110	0.4911	0.073*	
C19	0.55629 (10)	0.76409 (19)	0.3782 (3)	0.0533 (7)	
C20	0.59550 (10)	0.7442 (2)	0.2959 (3)	0.0551 (7)	
H20	0.6132	0.7884	0.2555	0.066*	
C21	0.60796 (10)	0.6603 (2)	0.2746 (3)	0.0547 (7)	
H21	0.6339	0.6478	0.2185	0.066*	
C22	0.50391 (11)	0.8728 (2)	0.4642 (5)	0.0783 (11)	
H22A	0.5006	0.9346	0.4656	0.117*	
H22B	0.5064	0.8514	0.5640	0.117*	
H22C	0.4765	0.8477	0.4121	0.117*	
O3'	0.6240 (3)	0.3830 (6)	0.1858 (9)	0.0605 (14)	0.37
N3'	0.6197 (5)	0.4734 (7)	0.2005 (15)	0.060 (2)	0.37
C12'	0.6199 (3)	0.2846 (5)	0.3878 (9)	0.0481 (11)	0.37
H12C	0.6000	0.2615	0.4631	0.058*	0.366 (4)
H12D	0.6467	0.3151	0.4360	0.058*	0.366 (4)
C13'	0.5914 (3)	0.3432 (4)	0.2836 (8)	0.0512 (10)	0.37
H13'	0.5647	0.3136	0.2302	0.061*	0.366 (4)
C14'	0.5746 (4)	0.4238 (4)	0.3791 (12)	0.0855 (13)	0.37
H14C	0.5400	0.4280	0.3741	0.103*	0.366 (4)
H14D	0.5858	0.4176	0.4821	0.103*	0.366 (4)

### Atomic displacement parameters (Å^2^)

**Table:**

	*U*^11^	*U*^22^	*U*^33^	*U*^12^	*U*^13^	*U*^23^
O1	0.125 (2)	0.0719 (16)	0.0621 (15)	−0.0375 (15)	0.0306 (14)	−0.0213 (12)
O2	0.0500 (13)	0.1008 (19)	0.0811 (16)	0.0140 (12)	0.0119 (11)	0.0077 (13)
O3	0.067 (3)	0.046 (2)	0.071 (3)	0.0145 (19)	0.034 (2)	0.000 (2)
O4	0.0630 (14)	0.0528 (13)	0.0976 (17)	0.0060 (10)	0.0090 (12)	−0.0198 (12)
N1	0.0545 (15)	0.0564 (15)	0.0630 (16)	−0.0168 (12)	0.0066 (12)	−0.0010 (12)
N2	0.0506 (14)	0.0556 (14)	0.0545 (14)	0.0116 (11)	0.0017 (11)	−0.0023 (11)
N3	0.067 (4)	0.045 (2)	0.072 (4)	0.010 (2)	0.033 (4)	0.0050 (19)
C1	0.0648 (19)	0.0446 (15)	0.0486 (16)	0.0190 (13)	0.0136 (14)	0.0038 (12)
C2	0.126 (3)	0.081 (3)	0.053 (2)	0.042 (2)	0.025 (2)	0.0108 (17)
C3	0.181 (6)	0.096 (4)	0.088 (3)	0.058 (4)	0.087 (4)	0.031 (3)
C4	0.127 (4)	0.076 (3)	0.160 (5)	0.042 (3)	0.110 (4)	0.053 (3)
C5	0.073 (2)	0.067 (2)	0.129 (4)	0.0121 (18)	0.052 (2)	0.033 (2)
C6	0.0553 (17)	0.0432 (15)	0.0664 (19)	0.0068 (13)	0.0247 (15)	0.0113 (13)
C7	0.068 (2)	0.075 (2)	0.075 (2)	0.0031 (18)	−0.0172 (17)	−0.0143 (18)
C8	0.0539 (18)	0.0577 (18)	0.0556 (17)	0.0165 (14)	0.0106 (14)	0.0099 (14)
C9	0.090 (2)	0.0436 (16)	0.066 (2)	0.0164 (16)	0.0442 (18)	0.0085 (14)
C10	0.079 (2)	0.0416 (16)	0.063 (2)	−0.0227 (15)	0.0292 (17)	−0.0111 (14)
C11	0.074 (2)	0.091 (3)	0.100 (3)	−0.034 (2)	−0.025 (2)	−0.004 (2)
C12	0.044 (3)	0.048 (2)	0.053 (3)	0.008 (2)	0.011 (2)	−0.0026 (19)
C13	0.056 (3)	0.044 (2)	0.055 (3)	0.0088 (19)	0.0169 (18)	−0.0011 (18)
C14	0.102 (3)	0.058 (2)	0.103 (3)	0.0323 (19)	0.063 (2)	0.0215 (18)
C15	0.079 (2)	0.0585 (19)	0.072 (2)	0.0267 (16)	0.0420 (18)	0.0198 (16)
C16	0.0589 (18)	0.0555 (17)	0.0531 (16)	0.0217 (14)	0.0234 (14)	0.0109 (13)
C17	0.075 (2)	0.0589 (19)	0.075 (2)	0.0219 (16)	0.0391 (18)	0.0160 (16)
C18	0.0598 (18)	0.065 (2)	0.0611 (19)	0.0216 (15)	0.0241 (15)	0.0025 (15)
C19	0.0533 (17)	0.0530 (17)	0.0531 (16)	0.0093 (14)	−0.0001 (13)	−0.0071 (13)
C20	0.0471 (16)	0.0578 (18)	0.0612 (18)	0.0004 (14)	0.0097 (14)	−0.0044 (14)
C21	0.0491 (16)	0.0656 (19)	0.0509 (17)	0.0133 (14)	0.0148 (13)	−0.0001 (14)
C22	0.0513 (18)	0.066 (2)	0.117 (3)	0.0156 (16)	−0.0008 (19)	−0.034 (2)
O3'	0.067 (3)	0.046 (2)	0.071 (3)	0.0145 (19)	0.034 (2)	0.000 (2)
N3'	0.067 (4)	0.045 (2)	0.072 (4)	0.010 (2)	0.033 (4)	0.0050 (19)
C12'	0.044 (3)	0.048 (2)	0.053 (3)	0.008 (2)	0.011 (2)	−0.0026 (19)
C13'	0.056 (3)	0.044 (2)	0.055 (3)	0.0088 (19)	0.0169 (18)	−0.0011 (18)
C14'	0.102 (3)	0.058 (2)	0.103 (3)	0.0323 (19)	0.063 (2)	0.0215 (18)

### Geometric parameters (Å, °)

**Table:**

O1—C10	1.222 (4)	C11—H11C	0.9600
O2—C8	1.226 (3)	C12—C13	1.502 (6)
O3—N3	1.407 (5)	C12—H12A	0.9700
O3—C13	1.451 (5)	C12—H12B	0.9700
O4—C19	1.364 (3)	C13—C14	1.531 (4)
O4—C22	1.437 (4)	C13—H13	0.9800
N1—C10	1.364 (4)	C14—C15	1.4965
N1—C6	1.426 (4)	C14—H14A	0.9700
N1—C11	1.470 (4)	C14—H14B	0.9700
N2—C8	1.354 (4)	C15—N3'	1.313 (8)
N2—C1	1.416 (4)	C15—C16	1.4638
N2—C7	1.469 (4)	C15—C14'	1.507 (7)
N3—C15	1.309 (5)	C16—C17	1.3846
C1—C6	1.394 (4)	C16—C21	1.390 (3)
C1—C2	1.392 (4)	C17—C18	1.379 (3)
C2—C3	1.356 (7)	C17—H17	0.9300
C2—H2	0.9300	C18—C19	1.376 (4)
C3—C4	1.371 (8)	C18—H18	0.9300
C3—H3	0.9300	C19—C20	1.395 (4)
C4—C5	1.393 (7)	C20—C21	1.360 (4)
C4—H4	0.9300	C20—H20	0.9300
C5—C6	1.394 (4)	C21—H21	0.9300
C5—H5	0.9300	C22—H22A	0.9600
C7—H7A	0.9600	C22—H22B	0.9600
C7—H7B	0.9600	C22—H22C	0.9600
C7—H7C	0.9600	O3'—N3'	1.410 (8)
C8—C9	1.508 (5)	O3'—C13'	1.442 (8)
C9—C10	1.521 (5)	C12'—C13'	1.503 (8)
C9—C12'	1.541 (7)	C12'—H12C	0.9700
C9—C12	1.564 (5)	C12'—H12D	0.9700
C9—H9	0.9800	C13'—C14'	1.602 (8)
C9—H9'	0.9800	C13'—H13'	0.9800
C11—H11A	0.9600	C14'—H14C	0.9700
C11—H11B	0.9600	C14'—H14D	0.9700
			
N3—O3—C13	109.0 (4)	O3—C13—C12	111.5 (4)
C19—O4—C22	117.1 (3)	O3—C13—C14	103.2 (3)
C10—N1—C6	122.7 (3)	C12—C13—C14	109.9 (3)
C10—N1—C11	117.8 (3)	O3—C13—H13	110.7
C6—N1—C11	119.2 (3)	C12—C13—H13	110.7
C8—N2—C1	122.6 (3)	C14—C13—H13	110.7
C8—N2—C7	117.9 (3)	C15—C14—C13	97.93 (17)
C1—N2—C7	118.8 (2)	C15—C14—H14A	112.2
C15—N3—O3	105.6 (5)	C13—C14—H14A	112.2
C6—C1—C2	118.9 (3)	C15—C14—H14B	112.2
C6—C1—N2	122.0 (2)	C13—C14—H14B	112.2
C2—C1—N2	119.0 (3)	H14A—C14—H14B	109.8
C3—C2—C1	120.7 (5)	N3—C15—C16	118.6 (3)
C3—C2—H2	119.6	N3'—C15—C16	125.8 (5)
C1—C2—H2	119.6	N3—C15—C14	115.7 (3)
C2—C3—C4	121.5 (5)	N3'—C15—C14	106.8 (5)
C2—C3—H3	119.3	C16—C15—C14	125.4
C4—C3—H3	119.3	N3'—C15—C14'	105.1 (6)
C3—C4—C5	119.0 (4)	C16—C15—C14'	126.8 (3)
C3—C4—H4	120.5	C17—C16—C21	117.81 (13)
C5—C4—H4	120.5	C17—C16—C15	121.1
C4—C5—C6	120.3 (5)	C21—C16—C15	121.05 (13)
C4—C5—H5	119.9	C18—C17—C16	121.65 (14)
C6—C5—H5	119.9	C18—C17—H17	119.2
C1—C6—C5	119.5 (3)	C16—C17—H17	119.2
C1—C6—N1	121.3 (2)	C17—C18—C19	119.5 (2)
C5—C6—N1	119.1 (3)	C17—C18—H18	120.3
N2—C7—H7A	109.5	C19—C18—H18	120.3
N2—C7—H7B	109.5	O4—C19—C18	124.9 (3)
H7A—C7—H7B	109.5	O4—C19—C20	115.5 (3)
N2—C7—H7C	109.5	C18—C19—C20	119.6 (3)
H7A—C7—H7C	109.5	C21—C20—C19	120.1 (3)
H7B—C7—H7C	109.5	C21—C20—H20	119.9
O2—C8—N2	122.0 (3)	C19—C20—H20	119.9
O2—C8—C9	121.9 (3)	C20—C21—C16	121.3 (2)
N2—C8—C9	116.0 (3)	C20—C21—H21	119.3
C8—C9—C10	105.4 (2)	C16—C21—H21	119.3
C8—C9—C12'	127.7 (4)	O4—C22—H22A	109.5
C10—C9—C12'	93.9 (4)	O4—C22—H22B	109.5
C8—C9—C12	104.2 (3)	H22A—C22—H22B	109.5
C10—C9—C12	120.5 (3)	O4—C22—H22C	109.5
C8—C9—H9	108.7	H22A—C22—H22C	109.5
C10—C9—H9	108.7	H22B—C22—H22C	109.5
C12'—C9—H9	110.0	N3'—O3'—C13'	107.8 (8)
C12—C9—H9	108.7	C15—N3'—O3'	117.6 (9)
C8—C9—H9'	109.2	C13'—C12'—C9	104.2 (5)
C10—C9—H9'	109.2	C13'—C12'—H12C	110.9
C12'—C9—H9'	109.2	C9—C12'—H12C	110.9
C12—C9—H9'	107.9	C13'—C12'—H12D	110.9
O1—C10—N1	122.1 (3)	C9—C12'—H12D	110.9
O1—C10—C9	121.9 (3)	H12C—C12'—H12D	108.9
N1—C10—C9	116.0 (3)	O3'—C13'—C12'	108.1 (7)
N1—C11—H11A	109.5	O3'—C13'—C14'	102.4 (6)
N1—C11—H11B	109.5	C12'—C13'—C14'	107.1 (7)
H11A—C11—H11B	109.5	O3'—C13'—H13'	112.9
N1—C11—H11C	109.5	C12'—C13'—H13'	112.9
H11A—C11—H11C	109.5	C14'—C13'—H13'	112.9
H11B—C11—H11C	109.5	C15—C14'—C13'	105.9 (5)
C13—C12—C9	110.4 (3)	C15—C14'—H14D	110.5
C13—C12—H12A	109.6	C13'—C14'—H14D	110.5
C9—C12—H12A	109.6	C15—C14'—H14C	110.5
C13—C12—H12B	109.6	C13'—C14'—H14D	110.5
C9—C12—H12B	109.6	H14C—C14'—H14D	108.7
H12A—C12—H12B	108.1		
			
C13—O3—N3—C15	−20.5 (6)	C12—C13—C14—C15	−144.4 (3)
C8—N2—C1—C6	51.7 (4)	O3—N3—C15—N3'	−69 (2)
C7—N2—C1—C6	−137.7 (3)	O3—N3—C15—C16	176.0 (3)
C8—N2—C1—C2	−130.1 (3)	O3—N3—C15—C14	2.1 (6)
C7—N2—C1—C2	40.5 (4)	O3—N3—C15—C14'	0.4 (8)
C6—C1—C2—C3	0.9 (5)	C13—C14—C15—N3	15.3 (4)
N2—C1—C2—C3	−177.3 (3)	C13—C14—C15—N3'	37.2 (8)
C1—C2—C3—C4	0.1 (7)	C13—C14—C15—C16	−158.1 (2)
C2—C3—C4—C5	−1.8 (7)	C13—C14—C15—C14'	66 (13)
C3—C4—C5—C6	2.5 (6)	N3—C15—C16—C17	179.2 (4)
C2—C1—C6—C5	−0.2 (4)	N3'—C15—C16—C17	154.3 (9)
N2—C1—C6—C5	178.0 (3)	C14—C15—C16—C17	−7.6
C2—C1—C6—N1	−179.0 (3)	C14'—C15—C16—C17	−5.8 (7)
N2—C1—C6—N1	−0.8 (4)	N3—C15—C16—C21	0.3 (4)
C4—C5—C6—C1	−1.5 (5)	N3'—C15—C16—C21	−24.6 (9)
C4—C5—C6—N1	177.4 (3)	C14—C15—C16—C21	173.52 (17)
C10—N1—C6—C1	−48.7 (4)	C14'—C15—C16—C21	175.2 (7)
C11—N1—C6—C1	137.5 (3)	C21—C16—C17—C18	−0.3 (2)
C10—N1—C6—C5	132.5 (3)	C15—C16—C17—C18	−179.28 (19)
C11—N1—C6—C5	−41.3 (4)	C16—C17—C18—C19	−0.6 (4)
C1—N2—C8—O2	175.7 (3)	C22—O4—C19—C18	−3.4 (4)
C7—N2—C8—O2	5.0 (4)	C22—O4—C19—C20	175.1 (3)
C1—N2—C8—C9	−7.3 (4)	C17—C18—C19—O4	179.3 (3)
C7—N2—C8—C9	−177.9 (3)	C17—C18—C19—C20	0.9 (5)
O2—C8—C9—C10	106.4 (3)	O4—C19—C20—C21	−178.8 (3)
N2—C8—C9—C10	−70.6 (3)	C18—C19—C20—C21	−0.2 (4)
O2—C8—C9—C12'	−1.3 (6)	C19—C20—C21—C16	−0.8 (4)
N2—C8—C9—C12'	−178.3 (4)	C17—C16—C21—C20	1.1 (3)
O2—C8—C9—C12	−21.4 (4)	C15—C16—C21—C20	−180.0 (2)
N2—C8—C9—C12	161.6 (3)	N3—C15—N3'—O3'	110 (3)
C6—N1—C10—O1	−178.6 (3)	C16—C15—N3'—O3'	−171.5 (8)
C11—N1—C10—O1	−4.8 (4)	C14—C15—N3'—O3'	−6.9 (14)
C6—N1—C10—C9	4.6 (4)	C14'—C15—N3'—O3'	−7.9 (15)
C11—N1—C10—C9	178.5 (3)	C13'—O3'—N3'—C15	12.3 (16)
C8—C9—C10—O1	−104.6 (3)	C8—C9—C12'—C13'	−87.0 (7)
C12'—C9—C10—O1	26.3 (5)	C10—C9—C12'—C13'	160.0 (6)
C12—C9—C10—O1	12.6 (4)	C12—C9—C12'—C13'	−44.5 (6)
C8—C9—C10—N1	72.1 (3)	N3'—O3'—C13'—C12'	−122.7 (10)
C12'—C9—C10—N1	−156.9 (4)	N3'—O3'—C13'—C14'	−9.9 (11)
C12—C9—C10—N1	−170.7 (3)	C9—C12'—C13'—O3'	−67.2 (8)
C8—C9—C12—C13	−172.4 (3)	C9—C12'—C13'—C14'	−176.8 (6)
C10—C9—C12—C13	69.8 (5)	N3—C15—C14'—C13'	−20.7 (10)
C12'—C9—C12—C13	41.0 (7)	N3'—C15—C14'—C13'	0.8 (12)
N3—O3—C13—C12	147.7 (5)	C16—C15—C14'—C13'	164.2 (4)
N3—O3—C13—C14	29.7 (5)	C14—C15—C14'—C13'	−151 (13)
C9—C12—C13—O3	54.9 (5)	O3'—C13'—C14'—C15	5.7 (10)
C9—C12—C13—C14	168.7 (3)	C12'—C13'—C14'—C15	119.3 (8)
O3—C13—C14—C15	−25.3 (3)		

### Hydrogen-bond geometry (Å, °)

**Table:**

*D*—H···*A*	*D*—H	H···*A*	*D*···*A*	*D*—H···*A*
C4—H4···O3^i^	0.93	2.51	3.367 (7)	154
C11—H11B···O1^ii^	0.96	2.56	3.501 (5)	168

Symmetry codes: (i) −*x*+3/2, −*y*+1/2, −*z*; (ii) −*x*+3/2, −*y*+1/2, −*z*+1.
